# Biosimilars in Pediatric Inflammatory Bowel Diseases: A Systematic Review and Real Life-Based Evidence

**DOI:** 10.3389/fphar.2022.846151

**Published:** 2022-03-17

**Authors:** Valeria Dipasquale, Giuseppe Cicala, Edoardo Spina, Claudio Romano

**Affiliations:** ^1^ Pediatric Gastroenterology and Cystic Fibrosis Unit, Department of Human Pathology in Adulthood and Childhood “G. Barresi”, University of Messina, Messina, Italy; ^2^ Department of Clinical and Experimental Medicine, University of Messina, Messina, Italy

**Keywords:** biosimilar, crohn’s disease, CT-P13, inflammatory bowel disease, pediatrics, safety, ulcerative colitis, anti-TNF-α

## Abstract

**Background:** Many pediatric inflammatory bowel disease (IBD) patients are now using biosimilars of anti-tumor necrosis factor-α (TNF-α), with increasing trends in recent years. This study reviewed all available data regarding the use of biosimilars in children with IBD.

**Methods:** PubMed, Google Scholar, Scopus, and CENTRAL databases were searched through keywords; inflammatory bowel diseases, Crohn’s disease, ulcerative colitis, biosimilar and child were combined using “AND” and “OR.” Original research articles involving pediatric patients receiving one of the biosimilar medications based on the anti-TNF-α biologic drugs approved for pediatric IBD treatment, independently from efficacy and drug response, were included.

**Results:** Nine studies were included in the evidence synthesis. CT-P13 was the biosimilar used in all studies. Four studies assessed the induction effectiveness of CT-P13. Clinical response and remission rates of biosimilar treatment were 86–90% and 67–68%, respectively, and they were not significantly different to the originator group. Five prospective studies on patients elected to switch from originator IFX to CT-P13 yielded similar results. Adverse events related to CT-P13 were mostly mild. The most frequently reported were upper respiratory tract infections. The switch from the originator had no significant impact on immunogenicity.

**Conclusion:** The current review showed reported CT-P13 effectiveness as measured by clinical response and/or remission rates after induction or during maintenance and suggest that there is no significant difference with that of the originator IFX. Further studies are warranted, including clinical, and pharmacovigilance studies.

## Introduction

Biologics were first introduced roughly 20 years ago, and have radically modified the treatment and prognosis of pediatric inflammatory bowel disease (IBD). Tumor necrosis factor-α (TNF-α), an inflammatory cytokine released by immune cells, was the target of the first biologics used to treat IBD patients (Laharie et al., 2005; Hyams et al., 2007; Hyams et al., 2012). The anti-TNF-α originator drugs available to treat IBD children are infliximab (IFX; Remicade©, Janssen) and adalimumab (Humira©, AbbVie). Their remarkable efficacy and safety profile has led to earlier (“top-down therapy”) and/or longer treatment duration, particularly in patients with a more severe course, and/or poor prognosis (Ruemmele et al., 2014; Turner et al., 2018). The patent on IFX expired in 2013, allowing the companies to launch its biosimilars. According to the World Health Organization, a biosimilar is defined as a “biotherapeutic product, which is similar in terms of quality, safety, and efficacy to an already licensed reference biotherapeutic product” (World Health Organization, 2009). CT-P13 was the first biosimilar IFX to be approved by the regulatory agencies, in 2013 by the European Medicine Agency (EMA) and in 2016 by the Food and Drug Administration (FDA) (Organization site European Medicines Agency, 2013; Organization site World Health Organization, 2017). IFX biosimilars are commercialized under different brand names, including Remsima© (Celltrion) and Inflectra© (Hospira) for CT-P13, or Flixabi© (Biogen) and Renflexis® (Merck) for SB2. ABP501 (Amgevita©, Amgen) was the first approved biosimilar to adalimumab. Based upon extrapolation of thorough *in vivo* experiments and two randomized controlled clinical studies in adult patients with rheumatologic diseases, biosimilars were authorized for the same indications as the original drug, including adult, and pediatric IBD (Park et al., 2013; Yoo et al., 2013; Alten and Cronstein, 2015). Extrapolation is the process of licensing a biosimilar for all the originator drug’s approved indications, even though the biosimilar has not been formally investigated in all the originator drug’s indications or populations (Weise et al., 2014; Alten and Cronstein, 2015; Vande Casteele and Sandborn, 2015). Extrapolation of compounds in the same class with the same mechanism of action from adult to pediatric or across indications is prevalent in clinical practice when there is insufficient evidence or clinical studies are underway (Weise et al., 2014; Vande Casteele and Sandborn, 2015). Adult patients have been the focus of studies evaluating the efficacy and safety of biosimilars in IBD (Farkas et al., 2015; Jahnsen et al., 2015; Gecse et al., 2016; Ye et al., 2019). According to the European Crohn’s and Colitis Organization’s (ECCO) guidelines, CT-P13’s effectiveness and safety are equivalent to those of its originator product drug in patients who are naïve to anti-TNF-α therapy or who have switched to CT-P13 (Danese et al., 2017). Many pediatric IBD patients are now using biosimilars, with growing trends in recent years. Data on the effectiveness and safety of biosimilars in pediatric IBD are steadily increasing (Dipasquale and Romano, 2020). CT-P13 can be regarded as a good alternative to the originator for induction and maintenance of remission in children with IBD, according to the European Society for Pediatric Gastroenterology, Hepatology, and Nutrition (ESPGHAN) Pediatric IBD Porto Group (de Ridder et al., 2015; de Ridder et al., 2019). A recent nationwide web survey conducted in Italy showed that most pediatric IBD experts have good knowledge about biosimilars, with awareness of similar efficacy and safety in comparison to the originator (Dipasquale et al., 2021).

The aim of this review was to analyze all the literature data, published after biosimilar use approval in 2013, regarding the use of biosimilars of anti-TNF-α in pediatric IBD patients, and to assess effectiveness, immunogenicity, and safety profiles, as well as cost concerns.

## Methods

### Search Strategy

Studies identification, screening, and extraction of relevant data were conducted according to the 2020 version of the Preferred Reporting Items for Systematic Reviews and Meta-Analyses (PRISMA) statement. Literature searches and screening of titles, abstracts and full text articles were conducted by two authors (VD; GC) independently. The research was conducted using the PubMed, Google Scholar, Scopus, and the Cochrane Central Register of Controlled Trials (CENTRAL) database—the latter also includes data from the clinicaltrials.gov and the World Health Organization International Clinical Trials Registry Platforms. Records provided by the academic search engine Google Scholar were also scanned. The considered timeframe for all scanned databases and searches was from 2013 to December 2021. For PubMed, Google Scholar and Scopus research, a query structure based on Boolean combinations of the terms “inflammatory bowel diseases,” “Crohn’s disease,” “ulcerative colitis,” “biosimilar” and “child,” with terms variations, was used. For Google scholar the search filter “only scientific articles” was also applied. For the complete query structure and the full list of filters and refinement used see Supplementary Box S1, S2. As for the CENTRAL database search, a multiple query strategy was used: a general query for IBDs, with Boolean combinations of the same terms used for other databases; two other queries of analogous structure to account for specific trials regarding Crohn’s disease and ulcerative colitis. For all the CENTRAL queries, the option of “search for word variations” was selected (full details are available in Supplementary Box S1). The references of all collected publications were also checked to find any missing relevant studies.

### Inclusion and Exclusion Criteria

Papers that fulfilled the following criteria were included: original research articles involving pediatric patients of any gender and ethnicity receiving one of the biosimilar medications based on the anti-TNF-α biologic drugs approved for pediatric IBD treatment, independently from efficacy and drug response. Studies were excluded if *1*) the originator drug only was used; *2*) biosimilars were used to treat diseases other than IBDs; *3*) articles were written in a language other than English.

### Data Extraction and Management

Data of relevance were extracted by a single author (V.D.) by the means of a data extraction sheet. Data regarding *1*) type of IBD treated, *2*) number of patients, *3*) type of biosimilar used, *4*) study duration, *5*) clinical evaluations, *6*) direct costs of treatment, were extracted. Missing data entries were marked with N/A (not available). Clinical response and/or remission as measured by the Pediatric Crohn’s Disease Activity Index (PCDAI) for CD or the Pediatric Ulcerative Colitis Activity Index (PUCAI) for UC were the primary outcomes. In most studies, clinical response was defined by a PCDAI drop of >15 and a PUCAI score of >20, while remission was defined by a PCDAI or a PUCAI score of 10 or less. No statistical analyses were performed due to the limited number of available studies and the heterogeneity in the reported data. Thus, the findings are presented in a descriptive manner.

## Results

**FIGURE 1:**
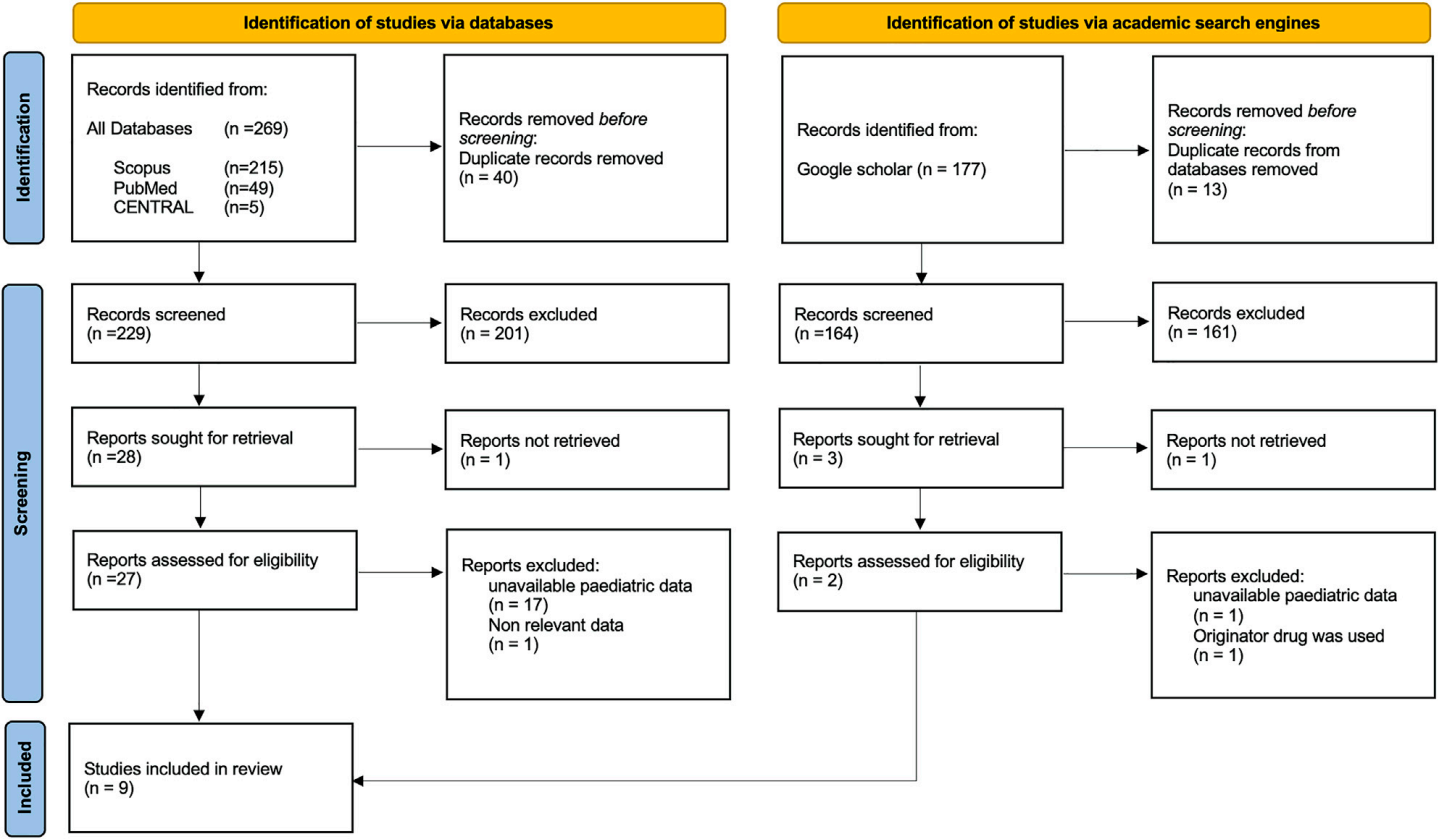
Flow chart for study retrieval and selection.

In total, 384 records were retrieved, 9 of which met the inclusion criteria (Figure 1) (Sieczkowska et al., 2016; Sieczkowska-Golub et al., 2017; Chanchlani et al., 2018; Gervais et al., 2018; Kang et al., 2018; Richmond et al., 2018; van Hoeve et al., 2019; Nikkonen and Kolho, 2020; Cheon et al., 2021). A total of 394 pediatric IBD patients (316 CD, 61 UC, and 17 IBD-U) was comprised. CT-P13 was the biosimilar used in all studies. No studies on other biosimilars of IFX (PF-06438179/GP1111, SB2) or adalimumab biosimilars in pediatric IBD have been performed so far.

### Clinical Endpoints

Each of the studies considered is summarized in Table 1. Some of them compared the outcomes with historical or reference cohorts. In most studies, patients received induction doses at 5 mg/kg at weeks 0, 2, and 6 (Sieczkowska-Golub et al., 2017; Kang et al., 2018; Richmond et al., 2018). In one study it was reported that 57% (16/28) of patients on CT-P13 received induction dose at > 5 mg/kg (Nikkonen and Kolho, 2020). The median age of included patients on CT-P13 was similar, ranging from approximately 11 to 14 years.

**TABLE 1:** Efficacy of biosimilars for pediatric inflammatory bowel disease.

References	Study design	Patients	Age, years^a^	Disease duration^b^	Controls	Time of assessment	Main outcomes
Sieczkowska-Golub J et al. (Sieczkowska-Golub et al., 2017)	Prospective	36 CD children, 27 anti-TNF naïve	11.79 ± 4.07	14 months (0.5–164)	No	Before the first and the fourth infusion (week 14)	86% (31/36) clinical response rate and 67% (24/36) remission rate
Richmond L et al. (Richmond et al., 2018)	Prospective	40 IBD children (29 CD, 11 UC)	12.7	12 months	No	At initiation and at week 12	67% (14/21) remission rate for CD patients
Chanchlani N et al. (Chanchlani et al., 2018)	Prospective	82 IBD children (63 CD, 14 UC, 5 IBD-U)	N/A	11.3 months (4.8–25.16)	175 (148 CD, 33 UC, 15 IBD-U) children on originator IFX	At initiation and at week 12	79% (19/24) and 68% (25/37) remission rates for biosimilar and originator IFX groups, respectively
Nikkonen A et al. (Nikkonen and Kolho, 2020)	Retrospective	28 IBD children (16 CD, 3 UC, 9 IBD-U)	12	13.2 months (0–87.6)	23 IBD children (17 CD, 2 UC, 4 IBD-U) on originator IFX	At initiation, at the third infusion, and at 1 year	90% clinical responses during induction with no difference between the two groups
							65 vs. 61% on maintenance treatment at 1 year (*p* > 0.05), respectively
Sieczkowska J et al. (Sieczkowska et al., 2016)	Prospective	39 IBD children (32 CD, 7 UC) elected to switch	11.1 ± 3.3 (CD) and 12.3 ± 2.3 (UC)	N/A	No	At switching (shortly before the first infusion), after the first and the second doses of biosimilar, and at the last follow-up assessment (mean 8 ± 2.6 months)	Statistically significant (*p* < 0.05) switching-related change in PCDAI
							88% (28/32) and 57% (4/7) clinical remission rate at the last follow-up assessment for CD and UC patients, respectively
Kang B et al. (Kang et al., 2018)	Prospective	38 IBD children (32 CD, 6 UC) elected to switch	14	N/A	36 IBD children (28 CD, 8 UC) on originator IFX	At switching (anytime during maintenance phase) and at 1 year	77.8% (28/36) and 78.9% (30/38) clinical remission rate for biosimilar and originator IFX groups, respectively
Gervais L et al. (Gervais et al., 2018)	Prospective	33 IBD children (26 CD, 4 UC, 3 IBD-U) elected to switch	11.8	N/A	No	Before the first dose of biosimilar, 6 and 12 months after switching	87% (25/31) and 83% (24/29) remission rates at 6 and 12 months, respectively
							No significant difference in remission rates within 12 months after switch
van Hoeve K et al. (van Hoeve et al., 2019)	Prospective	42 IBD children (26 CD, 16 UC) elected to switch	11.8	N/A	No	6 months before switching (baseline), at the last infusion before switching and 6 months after switching	83.3% (35/42) clinical remission rate 6 months after switching
							No significant difference in remission rates in comparison to baseline or at the last infusion before switch
Cheon JH et al. (Cheon et al., 2021)	Prospective	56 CD children (15 after switch)	N/A	N/A	No	At baseline, and at 6, 12, 24, 36, 42, and 48 months	Reduced PCDAI score at month 6 compared with baseline, remaining relatively consistent at most time points
							Lower proportion of PCDAI responders in the switch group

TNF, tumor necrosis factor; CD, Crohn’s disease; UC, ulcerative colitis; IBD, inflammatory bowel disease; IBD-U, inflammatory bowel disease unclassified; N/A not available; IFX, infliximab; PCDAI, Pediatric Crohn Disease Activity Index.

a At diagnosis.

b Before CT-P13 initiation.

#### Biosimilars as Primary Indication for anti-TNF-α

In a prospective Polish study, 36 pediatric CD patients were recruited from three institutions where the originator IFX was no longer accessible (Sieczkowska-Golub et al., 2017). CT-P13 treatment was indicated in the case of severe luminal CD and/or perianal disease that was resistant to standard treatment. Clinical response (a reduction of 12.5 points on the PCDAI) and remission (a PCDAI score of 10) were obtained in 86% and 67% of patients, respectively, at the end of the induction (week 14). No significant difference in remission rates between naïve and non-naive patients was found. The findings of this study were compared to those of the REACH study (Hyams et al., 2007), which established the efficacy and safety of the originator IFX, and identical clinical improvement and remission after three doses of biosimilar were shown. Other studies have shown similar remission rates (Chanchlani et al., 2018; Richmond et al., 2018). A prospective analysis of 278 IBD children from 27 UK sites found no differences in clinical response or remission rates after induction between the originator IFX (*n* = 82) and biosimilar IFX (*n* = 21) groups (Chanchlani et al., 2018). No significant difference in remission rates between the two groups was found. They were also compared new anti-TNF-α therapy patients to historical data from 398 patients who started on originator IFX in a prior United Kingdom IBD biologics audit (2011–2015) and they were found no significant differences in clinical response and remission rates at the same timepoint (Lynch et al., 2013; Russell et al., 2013). A retrospective Finnish study found that the originator IFX and biosimilar IFX therapies had similar first-year therapy outcomes, such as treatment intensification during follow-up (83 vs. 82%); treatment discontinuation during induction (8.7 vs. 3.6%) or follow-up (because of loss of response or adverse reaction; 39 vs. 36%); and treatment discontinuation due to anti drug antibodies (ADA) (17 vs. 3.4%) (Nikkonen and Kolho, 2020).

#### Biosimilars in Patients Switching From Originator anti-TNF-α

A total of 152 children (116 CD, 33 UC, and 3 IBD-U) were examined in five studies after switching from the originator IFX to CT-P13 (Sieczkowska et al., 2016; Gervais et al., 2018; Kang et al., 2018; van Hoeve et al., 2019; Cheon et al., 2021). In a prospective study, 39 IBD children were switched after (*n* = 37) or during (*n* = 2) induction (Sieczkowska et al., 2016). The effectiveness at the last biosimilar doses was assessed, and clinical remission rates for CD and UC patients were found to be 88% and 57%, respectively. Eighty percent of CD patients and all 4 UC patients who continued biosimilars at the last assessment visit (i.e., 11 months after the first patient had been switched, after a mean follow-up of 8 ± 2.6 months) were in remission (Sieczkowska et al., 2016). Later studies found similar results, with no clinically important changes in disease activity after switching. A prospective single-center study conducted in South Korea compared 38 IBD patients after the switch to CT-P13 with 36 patients remained on the originator IFX (Kang et al., 2018). Maintenance treatment of 1-year duration was continued by 86.1% of the patients on originator IFX, and 92.1% of those on biosimilar IFX. Eight patients did not complete the year of follow-up, because of complete remission (*n* = 3), loss of response and change to adalimumab (*n* = 3), and loss at follow-up (*n* = 2). Similar rates (77.8 vs. 78.9%) of sustained remission (i.e., 1 year of corticosteroid-free clinical remission with no further dose intensification) were observed in the two groups (Kang et al., 2018).

### Biomarkers Changes

Seven out of nine studies evaluated inflammatory biomarker changes (Supplementary Box). The Polish study evaluated C-reactive protein (CRP), erythrocyte sedimentation rate (ESR), platelets, and as well as hemoglobin (Hb) levels (Sieczkowska-Golub et al., 2017). CRP, ESR, and platelets had a significant reduction in all children who achieved a clinical response (Sieczkowska-Golub et al., 2017). More than half (59%) of individuals with elevated CRP levels at baseline had their CRP levels totally restored by week 14. In addition, one of the three children with anemia at week 0 had normalized Hb levels at week 14 (Sieczkowska-Golub et al., 2017). Similarly, Richmond et al. (Richmond et al., 2018) showed a significant decrease in CRP, ESR, and albumin serum levels at the end of induction with CT-P13. Studies investigating the switching from originator IFX to CT-P13 found no significant changes of inflammatory markers after switching (Sieczkowska et al., 2016; Gervais et al., 2018; Kang et al., 2018; van Hoeve et al., 2019). Fecal calprotectin was included in the analysis in four studies (Gervais et al., 2018; Kang et al., 2018; Richmond et al., 2018; Nikkonen and Kolho, 2020). Decreases were found to be not significant neither between baseline and follow-up visits, nor after switching from the originator IFX.

### Through Concentration

Five studies evaluated trough levels (TL) of IFX biosimilar (Table 2) (Gervais et al., 2018; Kang et al., 2018; Richmond et al., 2018; van Hoeve et al., 2019; Nikkonen and Kolho, 2020). Therapeutic trough values, when reported, were assessed to be in the range of 3–7 mg/L post-induction. When comparing CT-P13 patients to those on originator IFX, there were no significant differences in TL. Likewise, there was no substantial difference in TL changes after switching from originator IFX to CT-P13. Dose escalation or treatment intensification were used to optimize treatment for patients with subtherapeutic levels at baseline (Nikkonen and Kolho, 2020). Switching on immunogenicity has been examined in five pediatric studies (Gervais et al., 2018; Kang et al., 2018; Richmond et al., 2018; van Hoeve et al., 2019; Nikkonen and Kolho, 2020). After switching to the biosimilar CT-P13, it was not found any substantial increase in immunogenicity. When available, mean ADA levels did not differ substantially.

**TABLE 2:** Studies investigating trough levels.

References	Therapeutic range	Method	Time of assessment	Findings
Richmond L et al. (Richmond et al., 2018)	3–7 mg/L	N/A	Post-induction	Median TL 3.85 mg/L in 20/40 patients; level outside therapeutic range in 10/20
Nikkonen A et al. (Nikkonen and Kolho, 2020)	N/A	N/A	Third infusion (a)	Median TL 8.9 mg/L (originator group) and 14 mg/L (biosimilar group) (a)
			At any point (b)	TL < 2 mg/L in 61% of patients (originator group) and in 36% of patients (biosimilar group) (b)
				No significant difference between the two groups
Kang B et al. (Kang et al., 2018)	≥3 μg/ml	ELISA	1 year	Therapeutic TL in 90.3 and 88.6% of patients in originator and switch group, respectively
				No significant difference in therapeutic TL between the two groups
				No significant difference in therapeutic TL or median TL between baseline and 1-year follow up in the switch group
Gervais L et al. (Gervais et al., 2018)	3–7 mg/L	N/A	N/A	No significant changes in TL post-switch
van Hoeve K et al. (van Hoeve et al., 2019)	Lower limit 0.3 mcg/mL, upper limit 12 mcg/mL	ELISA	6 months before (baseline, a) and 6 months after switching (b)	Median TL 5.7 mcg/mL
				versus 6.5 mcg/mL (no significant difference)
				No significant difference between the proportion of patients with subtherapeutic levels at baseline or at the last infusion before switching and 6 months after

TL, trough level, ELISA enzyme-linked immunosorbent assay, N/A not available.

### Safety and Immunogenicity

Current available literature data reported only mildly to moderately severe adverse events (AEs) related to the IFX biosimilar. AEs related to IFX biosimilars in pediatric IBD patients were investigated in eight studies (Table 3) (Sieczkowska et al., 2016; Sieczkowska-Golub et al., 2017; Chanchlani et al., 2018; Gervais et al., 2018; Kang et al., 2018; Richmond et al., 2018; van Hoeve et al., 2019; Nikkonen and Kolho, 2020). In comparison to patients on originator IFX, CT-P13 patients had no significant differences in AE rates. Similarly, there was no significant difference when switching from originator IFX to CT-P13. Mild infections, predominantly upper respiratory tract infections, were the most commonly reported AEs. Three cases of Herpes zoster reactivation have been documented, one of which occurred after the first infusion of biosimilar IFX and necessitated therapy withdrawal (Sieczkowska et al., 2016). In seven cases, acute infusion reactions (AIRs) were observed, and in three of these, therapy was stopped (Sieczkowska et al., 2016; Sieczkowska-Golub et al., 2017; van Hoeve et al., 2019). During biosimilar treatment, one patient developed an ovarian teratoma (Sieczkowska et al., 2016). There was no information provided on demographics or disease progression. The patient had a total surgical ovary excision between consecutive biosimilar infusions. There was no need to adjust the dose. Cheon et al. (2021) found no additional safety findings in IBD patients treated with CT-P13 for up to 5 years, whether they were treated with or switched to CT-P13. In any case, there was no age-based subgroup analysis.

**TABLE 3:** Reported adverse events.

References	Premedication	AE	Discontinuation	ADA
Sieczkowska-Golub J et al. (Sieczkowska-Golub et al., 2017)	Yes	Upper respiratory tract infection (*n* = 6), AIR (*n* = 2), immediate raised blood pressure (*n* = 1), arthralgia (*n* = 1), Herpes simplex (*n* = 1), Herpes zoster (*n* = 1), pancreatitis (*n* = 1), suspected latent tuberculosis (*n* = 1)	*n* = 1 (AIR)	N/A
Richmond L et al. (Richmond et al., 2018)	N/A	AIR (*n* = 1)	Yes	Positive *n* = 2 at the end of the induction
Chanchlani N et al. (Chanchlani et al., 2018)	N/A	*n* = 2	N/A	N/A
Nikkonen A et al. (Nikkonen and Kolho, 2020)	N/A	Recurrent abscesses (n = 1)	Yes	Positive *n* = 2 at the end of the induction
Sieczkowska J et al. (Sieczkowska et al., 2016)	N/A	AIR (*n* = 3), upper respiratory tract infection (*n* = 7), viral diarrhea (*n* = 2), nausea, headache (*n* = 2), seborrhea (*n* = 1), epistaxis (*n* = 1), conjunctivitis (*n* = 1), pneumonia (*n* = 1), Herpes zoster (*n* = 1)	*n* = 2 (AIR, Herpes zoster)	N/A
Kang B et al. (Kang et al., 2018)	N/A	Upper respiratory tract infection (*n* = 10), acne (*n* = 4), hair loss (*n* = 3), aggravation of perianal fistula (*n* = 3), rash (*n* = 2), arthralgia (*n* = 1), leukopenia (*n* = 2), liver enzyme elevation (*n* = 1), headache (*n* = 1), Herpes zoster (*n* = 1), Norovirus infection (*n* = 1), viral conjunctivitis (*n* = 1)	No	Positive *n* = 2 at baseline, *n* = 2 at 12 months post-switch
Gervais L et al. (Gervais et al., 2018)	N/A	No significant AE reported; no AIR	No	Positive *n* = 16 at baseline, *n* = 8 at 6 months, *n* = 6 at 12 months post-switch
van Hoeve K et al. (van Hoeve et al., 2019)	N/A	AIR (*n* = 1), upper respiratory tract infections (*n* = 25), arthralgia (*n* = 5), gastroenteritis (*n* = 4), headache (*n* = 3), pharyngitis (*n* = 2), otitis media (*n* = 2), sinusitis (*n* = 1), conjunctivitis (*n* = 1), rash (*n* = 1)	*N* = 1 (AIR)	Positive *n* = 1 post-switch (not related to the AIR)

AE, adverse event; ADA, antidrug antibodies; AIR, acute infusion reaction.

### Costs

Three out of nine studies reported comparison of costs between originator IFX and CT-P13 (Table 4) (Chanchlani et al., 2018; Gervais et al., 2018; Richmond et al., 2018). All available data reported considerable cost reductions from using biosimilar IFX based on estimated and averaged local procurement rates.

**TABLE 4:** Cost saving in comparison to treatment with originator.

References	Drugs	Estimated saving	Time period
Richmond L et al. (Richmond et al., 2018)	Remsima© vs. Remicade©	38% average per phial	12 weeks
		£47,800 (€57,000) for the total number of infusions	
Chanchlani N et al. (Chanchlani et al., 2018)	Remsima© or Inflectra© vs. Remicade©	£875,000 (€998,526) for the total number of infusions	1 year
Gervais L et al. (Gervais et al., 2018)	Remsima© vs. Remicade©	£66,000 (€75,900)	1 year
		£1,500 per patient per year	

## Discussion

Overall, the findings of this systematic review article establish CT-P13 effectiveness as measured by clinical response and/or remission rates after induction or during maintenance and suggest that it does not significantly differ from that of the originator IFX.

Because the originator IFX is often no longer available, most IBD units have had to switch to or to start with its biosimilars (de Ridder et al., 2019; Dipasquale and Romano, 2020). Access to the originator IFX is also often limited due to the originator’s relatively expensive cost (de Ridder et al., 2019; Hughes et al., 2021). A growing number of children with IBD who have used biologics are being elected to switch to biosimilars (de Ridder et al., 2019; Dipasquale and Romano, 2020). Children with IBD were effectively transitioned from the originator IFX to CTP13 in available studies, without affecting the effectiveness, pharmacokinetics, immunogenicity, and safety (Sieczkowska et al., 2016; Gervais et al., 2018; Kang et al., 2018; van Hoeve et al., 2019). Switching occurred primarily during the maintenance phase and did not appear to be associated with a loss of efficacy over time, even in patients with mild-to-moderate disease activity. Single switches have been used in all studies. Following at least three induction infusions, ESPGHAN guidelines recommend transitioning to CT-P13 in IBD children in clinical remission (de Ridder et al., 2019). Because evidence on interchangeability is still sparse, multiple switches (>1 switch) between different biosimilars or between biosimilars and the originator are not currently advised (de Ridder et al., 2019). Switching to biosimilar IFX might raise the risk of immunogenicity, which is one of the key concerns about biosimilar usage in the pediatric IBD group. Loss of response, AEs, and delayed hypersensitivity responses are all linked to immunogenicity, as well as the formation of IFX ADA (Allez et al., 2010). All biologics have varying degrees of immunogenicity, and even modest variations in the formulation, purity, or packaging of a biological medication might impact its immunogenicity pattern (Gabbani et al., 2017). The findings of this systematic review suggest that biosimilars appear to be safe in pediatric IBD patients. Additionally, switching from the originator drug does not appear to raise immunogenicity considerably.

Biosimilars offer a more advantageous costing and reimbursement strategy, with price cuts ranging from 25 to 70% in Europe when compared to originator products (Brodszky et al., 2016). More than 90% of respondents in the previously cited Italian survey believed biosimilars to be cost-effective, with cost savings being the most important benefit of using biosimilars (Dipasquale et al., 2021). If the cost savings from the use of biosimilars were used to fund more biological treatments, several more IBD patients could be treated.

The extensive and systematic literature search is one of the strengths of this systematic review. The limitations largely reflect the shortcomings of the studies reviewed. First, they are observational studies reporting real-life data. Second, some of the included studies were limited with respect to sampling and generalizability. Moreover, the efficacy of biosimilars in the induction of mucosal healing was not investigated. Pediatric clinical trials and eventually more research into post-marketing surveillance data on effectiveness, safety, and immunogenicity are highly needed. Data on the efficacy and safety of adalimumab biosimilars in children with IBD are also warranted.

## Conclusion

More experiences regarding the effectiveness, immunogenicity, and interchangeability of biosimilars in pediatric IBD have been reported over the last few years. Their utilization has almost completely substituted that of the originator IFX due to greater availability and lower costs. There are no differences in efficacy and safety between originator IFX and CT-P13, according to current evidence. Nonetheless, regulatory legislation needs to be standardized, and more data on the interchangeability, pharmacokinetics, as well as pediatric specificities, are still desirable.

## Data Availability

The original contributions presented in the study are included in the article/Supplementary Material, further inquiries can be directed to the corresponding author.
