# Supplementary material for: Biosimilars in Pediatric Inflammatory Bowel Diseases: A Systematic Review and Real Life-Based Evidence
Source: Front Pharmacol. 2022 Mar 17;13:846151. doi: 10.3389/fphar.2022.846151 (PMC8970685; doi:10.3389/fphar.2022.846151)
Supplement: Supplementary file 1 [file DataSheet1.docx]

**Supplementary Box 1.** Detailed research queries and filters for all scanned databases

| **Pubmed** |
| --- |
| Research query:  (("inflammatory bowel diseases") OR ("Crohn’s disease") OR ("ulcerative colitis")) AND (biosimilar) AND ((child) OR (pediatrics)) AND ((infliximab) OR (adalimumab))  Filters:  Select only results from 2013 onward |
| **Scopus** |
| Research query + Refinements:  ( ( "inflammatory bowel diseases" ) OR ( "Crohn's disease" ) OR ( "ulcerative colitis" ) ) AND ( biosimilar ) AND ( ( child ) OR ( pediatrics ) ) AND ( ( infliximab ) OR ( adalimumab ) ) AND ( LIMIT-TO ( DOCTYPE , "ar" ) OR LIMIT-TO ( DOCTYPE , "ch" ) OR LIMIT-TO ( DOCTYPE , "ed" ) OR LIMIT-TO ( DOCTYPE , "no" ) ) AND ( LIMIT-TO ( LANGUAGE , "English" ) OR LIMIT-TO ( LANGUAGE , "Italian" ) ) AND ( LIMIT-TO ( PUBYEAR , 2022 ) OR LIMIT-TO ( PUBYEAR , 2021 ) OR LIMIT-TO ( PUBYEAR , 2020 ) OR LIMIT-TO ( PUBYEAR , 2019 ) OR LIMIT-TO ( PUBYEAR , 2018 ) OR LIMIT-TO ( PUBYEAR , 2017 ) OR LIMIT-TO ( PUBYEAR , 2016 ) OR LIMIT-TO ( PUBYEAR , 2015 ) OR LIMIT-TO ( PUBYEAR , 2014 ) ) AND ( LIMIT-TO ( SUBJAREA , "MEDI" ) OR LIMIT-TO ( SUBJAREA , "PHAR" ) OR LIMIT-TO ( SUBJAREA , "BIOC" ) OR LIMIT-TO ( SUBJAREA , "IMMU" ) )  Filters:  Already applied to the query using the “refinements” function |
| **CENTRAL** |
| Research queries:   1. (inflammatory bowel diseases):ti,ab,kw AND (pediatric):ti,ab,kw AND (Biosimilars):ti,ab,kw with Publication Year from 2013 to present, in Trials (Word variations have been searched) 2. (Crohn disease):ti,ab,kw AND (pediatric):ti,ab,kw AND (biosimilars): ti,ab,kw with Publication Year from 2013 to present, in Trials (Word variations have been searched) 3. (ulcerative colitis):ti,ab,kw AND (pediatric):ti,ab,kw AND (biosimilars): ti,ab,kw with Publication Year from 2013 to present, in Trials (Word variations have been searched)   Filters:  Already present in the queries |

**Supplementary Box 2.** Detailed research query and filters for academic search engine

| **Google Scholar** |
| --- |
| Research query:  (("inflammatory bowel diseases") OR ("Crohn’s disease") OR ("ulcerative colitis")) AND (biosimilar) AND ((child) OR (pediatrics)) AND ((infliximab) OR (adalimumab))  Filters:   - Select only results from 2013 onward - Select only scientific articles |

**Supplementary Box 3.** Studies investigating laboratory response

| Reference | Laboratory parameters | Findings |
| --- | --- | --- |
| Sieczkowska-Golub J et al. (23) | CRP, ESR, platelets, Hb | CRP, ESR, platelets significantly decreased/improved in patients with clinical response (no difference between naïve and non-naïve patients)  Hb increased (not significantly) |
| Richmond L et al. (24) | ﻿CRP, ESR, albumin, fecal calprotectin | Decreased/improved at week 12 (significantly) |
| Nikkonen A et al. (26) | Fecal calprotectin | Decreased; no difference between reference and biosimilar groups |
| Sieczkowska J et al. (27) | CRP, ESR, Hb | Decreased/improved |
| Kang B et al. (28) | White blood cell count, hematocrit, platelets, albumin, CRP, fecal calprotectin | ﻿Decreased/improved (not significantly) at 1 year; no difference between reference IFX and switch group |
| Gervais L et al. (29) | CRP, ESR, albumin, fecal calprotectin | Decreased (not significantly) 1 year after switch (not significantly) |
| van Hoeve K et al. (30) | ﻿CRP, ESR, platelets, albumin, Hb | CRP, ESR, albumin decreased/improved (not significantly) at 6 months  Platelets and Hb significantly increased |

CRP C-reactive protein, ESR erythrocyte sedimentation rate, Hb hemoglobin
